# Förster Resonance Energy Transfer Measurements of Ryanodine Receptor Type 1 Structure Using a Novel Site-Specific Labeling Method

**DOI:** 10.1371/journal.pone.0007338

**Published:** 2009-10-12

**Authors:** James D. Fessenden

**Affiliations:** Boston Biomedical Research Institute, Watertown, Massachusetts, United States of America; Temasek Life Sciences Laboratory, Singapore

## Abstract

**Background:**

While the static structure of the intracellular Ca^2+^ release channel, the ryanodine receptor type 1 (RyR1) has been determined using cryo electron microscopy, relatively little is known concerning changes in RyR1 structure that accompany channel gating. Förster resonance energy transfer (FRET) methods can resolve small changes in protein structure although FRET measurements of RyR1 are hampered by an inability to site-specifically label the protein with fluorescent probes.

**Methodology/Principal Findings:**

A novel site-specific labeling method is presented that targets a FRET acceptor, Cy3NTA to 10-residue histidine (His) tags engineered into RyR1. Cy3NTA, comprised of the fluorescent dye Cy3, coupled to two Ni^2+^/nitrilotriacetic acid moieties, was synthesized and functionally tested for binding to His-tagged green fluorescent protein (GFP). GFP fluorescence emission and Cy3NTA absorbance spectra overlapped significantly, indicating that FRET could occur (Förster distance = 6.3 nm). Cy3NTA bound to His_10_-tagged GFP, quenching its fluorescence by 88%. GFP was then fused to the N-terminus of RyR1 and His_10_ tags were placed either at the N-terminus of the fused GFP or between GFP and RyR1. Cy3NTA reduced fluorescence of these fusion proteins by 75% and this quenching could be reversed by photobleaching Cy3, thus confirming GFP-RyR1 quenching via FRET. A His_10_ tag was then placed at amino acid position 1861 and FRET was measured from GFP located at either the N-terminus or at position 618 to Cy3NTA bound to this His tag. While minimal FRET was detected between GFP at position 1 and Cy3NTA at position 1861, 53% energy transfer was detected from GFP at position 618 to Cy3NTA at position 1861, thus indicating that these sites are in close proximity to each other.

**Conclusions/Significance:**

These findings illustrate the potential of this site-specific labeling system for use in future FRET-based experiments to elucidate novel aspects of RyR1 structure.

## Introduction

The ryanodine receptor type 1 (RyR1) is an intracellular Ca^2+^ release channel that plays a central role in skeletal muscle excitation contraction coupling. This enormous homotetrameric protein embedded in the sarcoplasmic reticulum (SR) is part of a macromolecular complex that includes calmodulin, FK506 binding protein 12 kDa (FKBP12), the skeletal muscle voltage gated Ca^2+^ channel isoform (Cav1.1), and cyclic AMP dependent protein kinase [1]. RyR1 point mutations can result in human skeletal muscle disorders such as malignant hyperthermia, central core disease, and multiple minicore disease. Understanding the overall structure of this macromolecular complex and how perturbations of this structure by these mutations lead to skeletal muscle disease are central questions in skeletal muscle biology.

In cryo electron-microscopy (EM) reconstructions, the RyR is mushroom-shaped with four-fold symmetry reflective of the homotetrameric arrangement of the individual subunits [2]. Regions of the RyR implicated in muscle diseases have been localized to this cryo EM map via fusion of green fluorescent protein (GFP) into these elements and subsequent visualization of the corresponding increase in mass on the cryo EM map [3], [4]. In addition, changes in RyR structure that accompany channel gating have been determined by comparing cryo EM maps of the RyR locked in closed and open conformations [5], [6]. However, cryo EM techniques cannot resolve fast conformational changes of the RyR that occur as the channel gates in real-time nor can they be used to study RyRs in solution. Thus, additional methods are needed to perform these types of structural measurements of RyR1.

Förster resonance energy transfer (FRET) measurements are well-suited to examine real-time changes in RyR conformation and thus represent a novel approach to structure-based studies of RyR1. FRET relies upon energy transfer from a fluorophore in the excited state (donor) to a nearby photosensitive molecule (acceptor). The degree of energy transfer is directly dependent on donor-acceptor distances in the range of ∼2.5–10 nm and as such, this method is well-suited to make distance measurements on proteins in solution [7]. In addition, FRET can reliably measure changes in donor-acceptor distances that reflect the conformational dynamics of proteins.

FRET measurements require site-specific placement of donor and acceptor fluorophores onto the protein(s) being studied, often by covalent attachment at unique cysteine or lysine residues. However, for RyR1, site-specific fluorophore labeling at these residues is extremely difficult since this protein contains 100 cysteines and 229 lysines per subunit [8]. Thus, the first challenge in developing FRET methods to study RyR1 structure is to site-specifically label the protein with fluorophores suitable for FRET measurements. One novel approach to this problem has been to reconstitute RyR1 with fluorescently tagged RyR binding proteins, FKBP12 and calmodulin, and then to measure FRET between these proteins bound to RyR1 [9]. In this elegant study, distance measurements between these bound proteins were in close agreement with distances predicted from cryo EM localizations of their binding sites on RyR1 [10]. However, more versatile labeling methods are required to make FRET measurements between locations inaccessible to RyR-associated proteins.

Recently, a site-specific labeling method has been developed that holds great promise for use in FRET studies of RyR1. This method utilizes the interaction between nitrilotriacetic acid/Ni^2+^ (NTA) complexes and poly-histidine “tags” inserted into proteins for affinity purification [11] by coupling NTA to fluorescence acceptors [12] in order to target these molecules to His tags introduced into proteins. Using this method, the structures of the serotonin receptor [13], the CAP DNA-binding protein [12] and the NK1 tachykinin receptor [13] have been mapped using FRET. NTA has also been coupled with quantum dots [14], biotin [15], and conformationally sensitive fluorophores [16] to site-specifically attach these substances to His-tagged proteins.

In the present study, this site-specific labeling method has been adapted for labeling RyR1 with Cy3, which can then accept fluorescence energy from GFP fused into the RyR1 primary sequence. This study outlines the procedures for synthesis and testing of the Cy3NTA labeling reagent as well as the construction of His-tagged GFP-RyR1 fusion proteins suitable for FRET. In addition, this study details FRET measurements in intact RyRs using this site-specific labeling method.

## Methods

### Cy3NTA Synthesis and Purification

**Figure 1:**
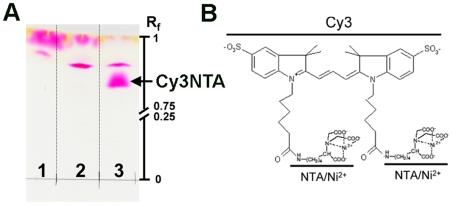
TLC analysis and structure of Cy3NTA. (A) Products from reactions consisting of Cy3-bis-succinimidyl ester alone (lane 1), Cy3-mono-succinimidyl ester and AB-NTA (lane 2), and Cy3-bis-succinimidyl ester and AB-NTA (lane 3) separated by TLC as described in Methods. Arrow indicates Cy3NTA used for FRET studies. R_f_ = relative index of mobility. (B) Predicted structure of Cy3NTA. Locations of Cy3 and the two NTA/Ni^2+^ moieties are indicated.

Cy3NTA was synthesized via incubation of 50 mM N-(5-amino-1-carboxypentyl)iminodiacetic acid (Dojindo Molecular Technologies, Rockville, MD) with 1 mM Cy3-bis-succinimidyl ester (GE Healthcare Biosciences Corp., Piscataway, NJ) in 0.2 M NaCO_3_ pH 9.0 buffer for 1 hr at room temperature in the dark. The reaction mixture was then spotted on a silica gel plate and resolved by thin layer chromatography (TLC) using a solvent system comprised of 33∶21∶6 NH_4_OH:ethanol:water [12]. The TLC plate was dried and examined directly for products (Fig. 1). Cy3NTA (R_f_ = 0.81) was scraped and extracted with 60% methanol. Yield as determined spectrophotometrically (Cy3 ε_550_ = 150,000 M^−1^ cm^−1^) was typically 40% of starting material. NTA conjugation affected neither absorption nor fluorescence emission of Cy3 (data not shown). Cy3NTA was dried and stored at –80 C in individual aliquots each containing 10 nmoles of product. Before use, the two NTA groups of Cy3NTA were charged with 20 nmoles of NiCl_2_ in water to form a stock solution comprised of 500 µM Cy3NTA and 1 mM NiCl_2_ used for Cy3NTA titrations in functional assays.

### GFP Synthesis

Oligonucleotides encoding His_6_ or His_10_ tags were inserted into the 5′ end of the cDNA encoding GFP from *Aequorea coerulescens* (AcGFP; Takara BIO, Mountain View, CA) using standard molecular biology techniques. GFP was expressed in OneShotBL21Star(DE3) *E. Coli* bacteria (Invitrogen, Carlsbad, CA) after induction with 0.4 mM isopropyl thiogalactoside. Bacterial lysates obtained by sonication on ice in a buffer containing 50 mM Na_2_HPO_4_, 300 mM NaCl, pH 8.0 were incubated at 4C for 3 days to allow complete maturation of the GFP chromophore.

### *In vitro* GFP Fluorescence Measurements

*In vitro* measurements of GFP fluorescence were performed on a LS55 luminescence spectrometer (PerkinElmer, Waltham, MA) in buffer consisting of 50 mM Na_2_HPO_4_, 300 mM NaCl, pH 8.0 in a stirred quartz microcuvette at room temperature. GFP fluorescence was observed via excitation of the sample with 475 nm light and monitoring emission at 510 nm. Raw fluorescence data was corrected for dilution and inner filter effect and then expressed as fraction of initial fluorescence (i.e. F/F_0_).

Cy3NTA titration data was fitted using:
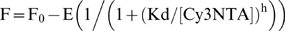
to determine F_0_ (initial GFP fluorescence), h (Hill coefficient), Kd (dissociation constant), and E (level of FRET between GFP and Cy3).

### Förster Distance Calculations

**Figure 2:**
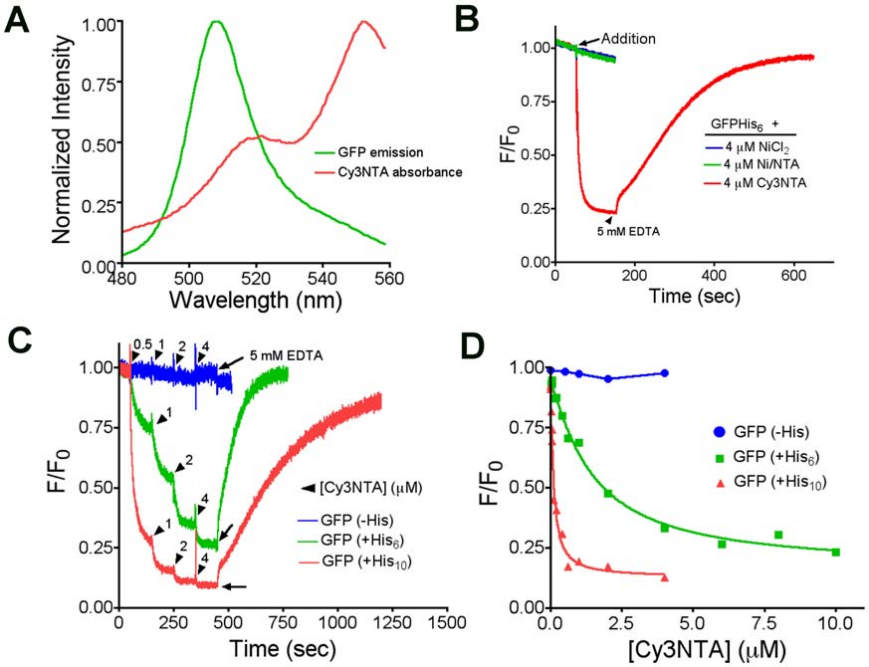
Cy3NTA quenches fluorescence of a His-tagged GFP. (A) Normalized GFP emission (green, λ_Ex_ = 476 nm) and Cy3NTA absorbance (red) spectra. (B) Time-based measurements of GFPHis_6_ fluorescence normalized to initial intensity (F_0_). Arrow indicates point of addition of either 4 µM NiCl_2_ (blue trace), Ni/NTA (green) or Cy3NTA (red). Arrowhead indicates addition of 5 mM EDTA. (C) Time-based fluorescence measurements of GFP either lacking (-His; blue trace) or containing an N-terminal His tag with either 6 (green) or 10 (red) residues. Arrowheads indicate additions of Cy3NTA at the (µM) concentrations indicated. Arrow indicates addition of 5 mM EDTA. (D) Detailed fluorescence quenching curves for Cy3NTA binding to each of the 3 GFP constructs. Values are averages of GFP fluorescence after addition of Cy3NTA. Quenching data for His-tagged GFP constructs were fit as described in Methods.

The Förster distance (at which 50% energy transfer occurs) between GFP and Cy3 was calculated from the degree of overlap between the GFP emission spectrum and Cy3 absorption spectrum (Fig. 2a) using:
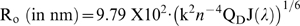
where the orientation factor, κ^2^ = 0.66 (assuming random orientation of the donor and acceptor dipoles), refractive index of the medium (assumed to be water) between donor and acceptor, *n* = 1.4, quantum yield of GFP donor Q_D_ = 0.79 and overlap integral J(λ) = 5.21×10^−13^ M^−1^ cm^3^ calculated using:
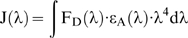
where ε_A_(λ) and F_D_(λ) represent the molar extinction coefficient of the acceptor (in cm^−1^ M^−1^) and the normalized donor emission (in %) at wavelength λ (in cm), respectively.

### RyR1 cDNA Cloning

Primers containing *San*D I sites were used to amplify cDNA encoding AcGFP using polymerase chain reaction (PCR). The 3′ primer also encoded a glycine rich segment: GGGGSGGGG. The resulting 0.75 kB product cloned into TOPO TA vector (Invitrogen) was confirmed using DNA sequencing. After cleavage with *San*D I, the AcGFP cDNA was inserted into a unique *San*D I restriction site introduced at the 5′ end of the rabbit RyR1 cDNA to create GFP-RyR1(-His). To create GFP(618)-RyR1, primers containing *Bgl* II sites that each encoded the glycine rich segment indicated above were used to amplify cDNA encoding AcGFP using PCR. After cloning into TOPO TA and sequence verification, the AcGFP cDNA was inserted into a naturally occurring *Bgl* II site corresponding to amino acid position 618 in the RyR1 primary sequence. Primers encoding His_10_ tags at either the 5′ or 3′ end of GFP were used to amplify AcGFP and the resulting product was cloned into the *San*D I site at amino acid position 1 to create GFP-RyR1 (N-term His) and GFP-RyR1 (1′His) respectively. A linker encoding a His_10_ tag was inserted into a naturally occurring *Bgl* II site corresponding to amino acid position 1861 to create GFP-RyR1 fusion proteins with a His_10_ tag at this site.

### Cell Culture and Transfection

HEK-293T cells were grown in Dulbecco's modified Eagles medium supplemented with 10% fetal calf serum (Hyclone), 2 mM L-glutamine, 100 units/ml penicillin, and 100 µg/ml streptomycin sulfate. Cells were transfected with cDNAs encoding GFP-tagged RyR1 proteins as follows: A solution consisting of 10 µg of cDNA in 300 µl of DMEM supplemented with 0.1 mg/ml linear polyethylenimine (Polysciences Inc., Warrington, PA) incubated for 15 min at room temperature was added to 500,000 HEK293T cells. After 24 hours, cells were transferred to 16-well slides (Nunc, Rochester, NY) precoated with ECM reagent (Sigma Aldrich, St. Louis, MO) diluted 1∶2 in DMEM and cells were then utilized for experiments 24 hr later.

### Ca^2+^ Imaging

HEK-293T cells expressing RyRs were loaded with 5 µM Fura-2 AM (Invitrogen) for 30 min at 37C in “imaging buffer” consisting of 125 mM NaCl, 5 mM KCl, 1.2 mM MgSO_4_, 2 mM CaCl_2_, 6 mM glucose and 25 mM HEPES, pH 7.4. After washing and a further 30 min incubation at 37C to complete de-esterification of Fura-2, cells were imaged at 40× magnification using a Zeiss Axiovert 200 epifluorescence microscope (Thornwood, NY) interfaced with a Hyperswitch Ca^2+^ imaging system (Ionoptix Corp., Milton, MA). Cells were perfused with a graded series of caffeine concentrations in imaging buffer using a Valvebank 8-channel perfusion system (Automate Scientific, Berkeley, CA) and changes in intracellular Ca^2+^ were recorded as changes in Fura-2 fluorescence ratio (Emission = 510 nm) when the cells were alternately excited at 340 and 380 nm at 60 Hz.

Normalized Ca^2+^ transients were quantified from the area under each transient using Ionoptix analysis software. This data plotted as a function of caffeine concentration was fit to a sigmoidal dose-response function (variable slope) to determine EC_50_ values that were compared using 1-way analysis of variance (ANOVA) using Prism 4 software (Graphpad Inc., San Diego, CA).

### FRET Imaging

HEK-293T cells expressing GFP-RyR1 fusion proteins were permeabilized for 30 min at room temperature in “FRET buffer” comprised of 125 mM NaCl, 5 mM KCl, 6 mM glucose, 25 mM HEPES pH 7.6 supplemented with 0.02% saponin. Cells were imaged on a Leica TCS SP5 confocal microscope (Mannheim, Germany) via illumination at 476 nm and recording GFP and Cy3 fluorescence using PMT windows set at 500–525 nm and 550–610 nm, respectively. Image data was recorded as a series of confocal Z-stacks comprised of 30 images recorded over a 100 µm distance. Fluorescence for a given cell was quantified from the peak fluorescence value recorded from a region of interest placed on that cell. GFP fluorescence recorded 60 min after addition of Cy3NTA was normalized to resting fluorescence to determine F/F_0_ values for each cell which were then averaged for all cells at each [Cy3NTA]. Data plotted as a function of [Cy3NTA] was fit as described for the Ca^2+^ imaging data (see above). Energy transfer efficiency (E) was calculated using
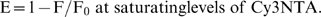



### NTA-agarose Column Chromatography

HEK-293T cells expressing GFP-RyR1 fusion proteins (∼500,000 total cells) were sonicated in FRET buffer supplemented with a commercial mixture of common protease inhibitors (Roche Applied Sciences, Mannheim, Germany) lacking divalent metal ion chelators. The resulting lysates were fractionated on a 0.2 ml NTA-agarose column (Qiagen, Valencia, CA) pre-equilibrated in FRET buffer. Individual 150 µl fractions were collected in a 96-well polystyrene plate after column washes with buffer alone, followed by FRET buffer containing 30 mM imidazole and 300 mM imidazole.

After a 1 hr incubation to allow protein adsorption, RyR content in individual fractions was quantified as follows. Plates were washed with “TBS-T buffer” consisting of 50 mM Tris-HCl, 150 mM NaCl, 0.1% Tween-20 pH 7.4 and then blocked with 5% nonfat milk in TBS-T for 1 hr at RT. After washing with TBS-T, plates were incubated 1 hr in 34C anti-RyR primary antibody (Developmental Studies Hybridoma Bank, Iowa City, IA) diluted 1∶200 in TBS-T, followed by washing in TBS-T and 1 hr incubation in goat anti-mouse HRP conjugated secondary antibody (Invitrogen) diluted 1∶1000 in TBS-T. After washing, plates were incubated in TMB substrate (Thermo Scientific, Rockford, Il) followed by neutralization with 0.15 N H_2_SO_4_. TMB absorbance (λ_max_ = 450 nm) was quantified on a Tecan Safire II plate reader (Grodig, Austria).

## Results

### Synthesis and Purification of Cy3NTA

TLC analysis was used to examine reaction products between Cy3-succinimidyl esters and N-(5-amino-1-carboxypentyl)iminodiacetic acid (AB-NTA) (Fig. 1a). Cy3-**bis**-succinimidyl ester alone (Bis-Cy3) ran at the solvent front (lane 1) whereas incubation of Cy3-**mono**-succinimidyl ester with AB-NTA resulted in an additional product migrating at R_f_ = 0.86 (lane 2). Incubation of Cy3-**bis**-succinimidyl ester with AB-NTA (lane 3) resulted in an additional predominant product migrating at R_f_ = 0.81 that was purified and reconstituted with Ni^2+^ to form the FRET acceptor, Cy3NTA (Fig. 1b).

### Functional Ttesting of Cy3NTA

To determine if Cy3NTA could act as a His-tag specific FRET acceptor, *in vitro* fluorimetry was used to determine the ability of this compound to quench fluorescence of His-tagged GFP via FRET. Overlap between the emission spectrum of GFP and the absorbance spectrum of Cy3NTA (Fig. 2a) was used to calculate a predicted Förster distance (at which 50% energy transfer occurs) of 6.30 nm for this donor-acceptor pair. Incubation of His_6_-tagged GFP with 4 µM Cy3NTA resulted in a 75% decrease in GFP fluorescence that was completely reversed by 5 mM EDTA (Fig. 2b). Incubation of GFPHis_6_ with either 4 µM Ni^2+^ or 4 µM Ni^2+^/NTA did not enhance GFP quenching (Fig. 2b) and no GFP quenching was observed in the presence of Cy3 alone or when Cy3NTA was added in the presence of 150 mM imidazole (data not shown). While Cy3NTA did not quench fluorescence of GFP lacking a His tag (Fig. 2c; blue trace), Cy3NTA did quench GFP containing a His_6_ tag in a concentration dependent manner that could be reversed by EDTA (Fig. 2c; green trace). Lengthening the His tag to 10 residues enhanced the degree of quenching and the apparent affinity of Cy3NTA binding (red trace). The concentration dependence of Cy3NTA quenching of GFPHis_6_ or GFPHis_10_ fluorescence (Fig. 2d) yielded apparent affinities of 1.01 and 0.11 µM, respectively and degrees of quenching of 72% and 88% respectively.

Levels of Ni^2+^/NTA up to 100 µM had no effect on RyR1 activity, as indicated by [^3^H]ryanodine binding analysis of heavy SR vesicles from skeletal muscle (data not shown). This data suggests that Ni^2+^, when complexed with NTA, does not affect RyR1 function.

### Construction, Expression and Functional Testing of His-Tagged GFP-RyR1 Fusion Proteins

**Figure 3:**
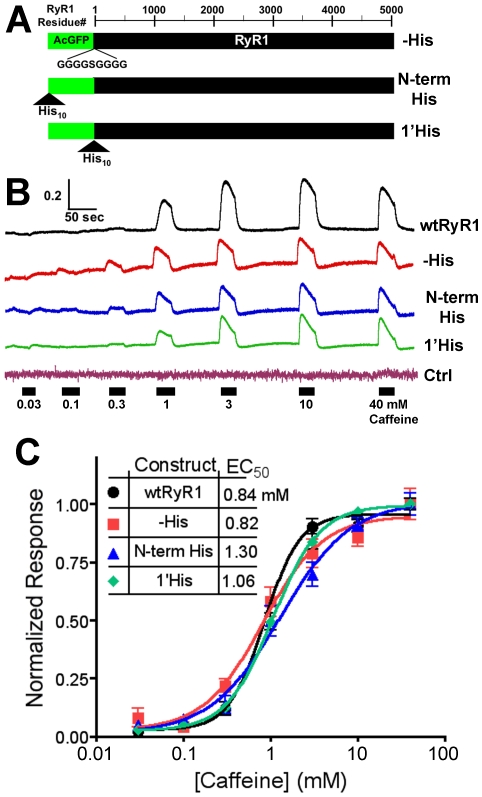
GFP-RyR1 fusion proteins release Ca^2+^ in response to caffeine stimulation. (A) GFP-RyR1 fusion constructs tested for FRET. Bars represent amino acid sequence of GFP (green) and RyR1 (black). The sequence of the glycine rich linker between GFP and RyR1 as well as positions of inserted His_10_ tags are shown. Scale indicates RyR1 amino acid number. (B) Caffeine-induced Ca^2+^ transients in HEK-293T cells expressing RyR1 constructs measured using Fura-2 based Ca^2+^ imaging. A graded series of caffeine concentrations were perfused at the times and concentrations indicated (black bars). Individual representative traces indicate changes in Fura-2 fluorescence excitation F340/F380 ratio. Ctrl indicates untransfected cells. Calibration bar = 0.2 340/380 ratio units vs. 50 s. (C) Normalized caffeine dose response curves for the indicated constructs. Values represent mean +/− S.E.M. for 19–22 cells per construct. EC_50_ values were calculated from the midpoint of these curves as described in Methods. No statistical difference (1-way analysis of variance (ANOVA); p<0.05) between EC_50_ values was observed.

To determine if Cy3NTA could be used for FRET-based structural measurements of RyR1, GFP was fused to the N-terminus of RyR1 to act as a fluorescence donor. Then, three GFP-RyR1 constructs were created (Fig. 3a) that either lacked a His tag (-His) or contained a His_10_ tag at either the N-terminus (N-term His) or between the fused GFP and RyR1 (1′His). Expression of these constructs in HEK-293T cells was verified via confocal microscopy by observing GFP fluorescence that co-localized with anti-RyR immunoreactivity (data not shown). The function of these fusion proteins was tested using Fura-2 based Ca^2+^ imaging (Fig. 3b). The RyR activator, caffeine, triggered Ca^2+^ release in HEK-293T cells expressing either wtRyR1 or each of the 3 GFP-RyR1 fusion proteins whereas untransfected cells did not respond to caffeine. The EC_50_ for caffeine activation for each GFP-RyR1 fusion construct was unchanged compared to wildtype RyR1 (Fig. 3c).

**Figure 4:**
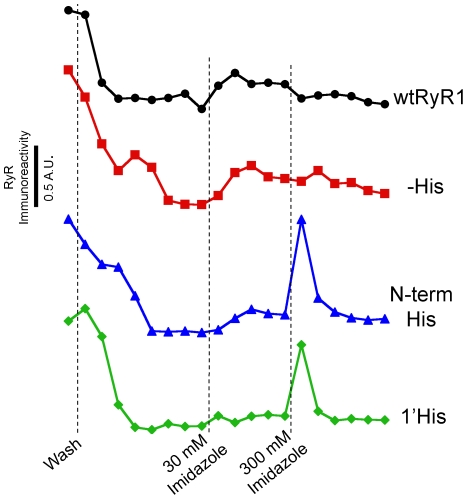
His-tagged GFP-RyR1 fusion proteins bind to NTA-agarose. NTA-agarose-based fractionation of crude lysates from HEK-293T cells expressing GFP-RyR1 fusion proteins. Columns were washed as indicated (dotted lines). Datum points indicate relative levels of RyR immunoreactivity in consecutive 120 µl fractions quantified by an RyR-specific ELISA assay as described in Methods.

The inserted His_10_ tags must be present on the surface of RyR1 in order for Cy3NTA to bind. The surface exposure of the inserted His_10_ tags was determined using NTA-agarose affinity chromatography (Fig. 4). Lysates prepared from HEK-293T cells expressing these RyR constructs were fractionated on an NTA-agarose column washed with 30 mM imidazole (to elute weakly bound proteins) and 300 mM imidazole (to elute tightly bound proteins). Both His-tagged GFP-RyR1 fusion proteins eluted with 300 mM imidazole, thus indicating that these constructs bound tightly to the column. In contrast, neither wtRyR1 nor GFP-RyR1(–His) were eluted with 300 mM imidazole thus suggesting that these proteins did not bind tightly to the column.

### FRET Measurements of His-Tagged GFP-RyR1 Fusion Proteins Using Cy3NTA

**Figure 5:**
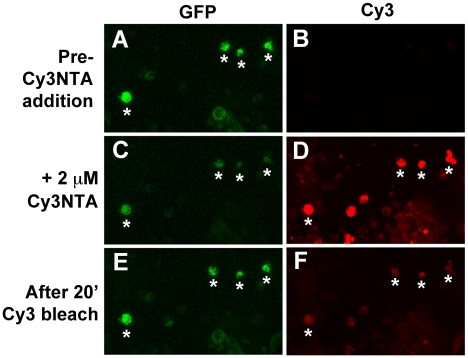
Cy3NTA quenches GFP-RyR1 (N-term His) fluorescence via FRET. HEK-293T cells expressing GFP-RyR1 (N-term His) were examined for GFP (panels A,C,E) and Cy3 fluorescence (B,D,E) either before (A,B) or after (C,D) a 30 min incubation with 2 µM Cy3NTA. Cy3 fluorescence was observed both in HEK-293T cells expressing GFP-RyR1(N-term His) (asterisks) and untransfected cells. Cy3 was then selectively bleached via 20 min illumination with 550 nm light resulting in an increase in GFP fluorescence (panel E) and a decrease in Cy3 fluorescence (F).

The ability of Cy3NTA to quench fluorescence of GFP-RyR1(N-term His) expressed in HEK-293T cells was determined using confocal microscopy (Fig. 5). After incubation with 2 µM Cy3NTA, GFP-RyR1(N-term His) fluorescence decreased by 75%, whereas Cy3 fluorescence appeared in both transfected and untransfected cells. After photobleaching Cy3 for 20 min, GFP-RyR1(N-term His) fluorescence recovered to 85% of its initial level.

**Figure 6:**
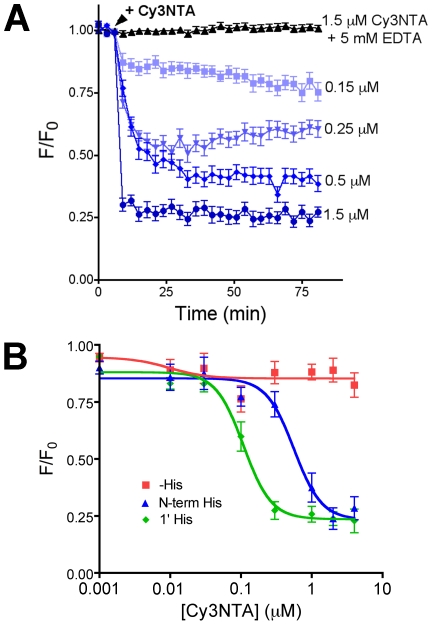
Cy3NTA quenches His-tagged GFP-RyR1 fluorescence in a concentration-dependent manner. (A) Concentration dependence of Cy3NTA quenching of GFP-RyR1 (N-term His) fluorescence. The indicated Cy3NTA concentrations were added to HEK-293T cells expressing GFP-RyR1 (N-term His) at the time point indicated (arrowhead) and the change in GFP fluorescence normalized to initial fluorescence (F/F_0_) was measured. Pre-incubation with 5 mM EDTA (top trace) abolished fluorescence quenching by Cy3NTA. (B) Cy3NTA concentration dependence of quenching of the indicated GFP-RyR1 fusion proteins expressed in HEK-293T cells. Values represent mean +/− S.E.M. for 7–14 cells. EC_50_ and FRET efficiency values were determined from these curves as described in Methods.

GFP-RyR1(N-term His) fluorescence was quenched in a concentration dependent manner by Cy3NTA that could be blocked by preincubation with 5 mM EDTA (Fig. 6a) or with 150 mM imidazole (data not shown). Cy3NTA quenched GFP-RyR1(N-term His) and GFP-RyR1(1′His) to similar degrees (76.7% and 76.5% respectively) whereas this compound quenched GFP-RyR1(-His) by only 14.6% (Fig. 6b). The apparent affinity for Cy3NTA binding to GFP-RyR1(N-term His) and GFP-RyR1(1′ His) was 0.56 and 0.11 µM, respectively.

**Figure 7:**
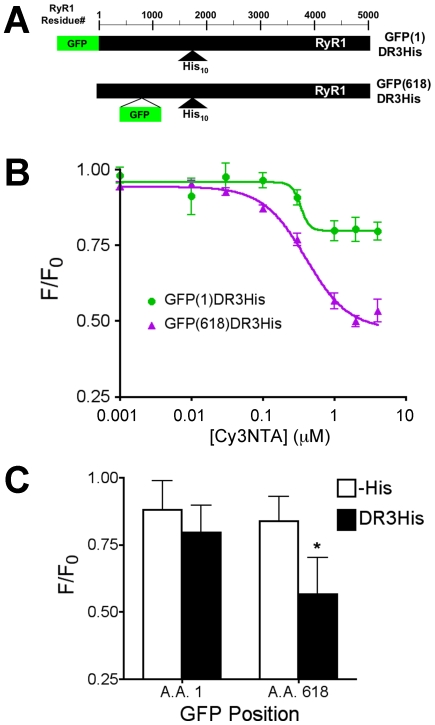
Cy3NTA can be used to measure FRET between internal sites on RyR1. (A) GFP-RyR1 fusion constructs containing a His_10_ tag in divergent region 3 at amino acid residue 1861. Bars represent amino acid sequence of GFP (green) and RyR1 (black). For construct GFP(618)DR3His, GFP inserted at amino acid residue 618 was flanked by poly-glycine segments as indicated in Methods. Scale indicates RyR1 amino acid number. (B) Cy3NTA concentration-dependence of quenching of GFP(1)DR3His (green trace) and GFP(618)DR3His (purple) expressed in HEK-293T cells. Data was fit to determine EC_50_ and the level of energy transfer (E) as described in Methods. (C) Degree of quenching of the indicated GFP-RyR1 fusion proteins by 1 µM Cy3NTA. Asterisk indicates significant level of quenching (p<0.01) relative to GFP-RyR1 (-His) as determined using 1-way ANOVA followed by a Dunnett's post test.

To determine if this system could be used to measure FRET between internal sites of RyR1, additional fusion proteins were created that contained a His_10_ tag at amino acid residue 1861 in divergent region 3 (DR3) [17] and GFP at either the N-terminus (to create construct GFP(1)DR3His) or at amino acid position 618 (to create construct GFP(618)DR3His (Fig. 7a)). These constructs could be activated by caffeine similar to wtRyR1 in Ca^2+^ imaging experiments and the His_10_ tag was surface exposed as determined using NTA-agarose column chromatography (data not shown). Cy3NTA quenched GFP(1)DR3His to a similar extent (20.1%) compared to GFP-RyR1(-His) whereas Cy3NTA quenched GFP(618)DR3His by 52.7% (Fig. 7b). Comparison of quenching by 1 µM Cy3NTA of GFP-RyR1 fusion constructs with GFP at either position 1 or 618 that lacked or contained a His_10_ tag at DR3 indicated significant levels of quenching only for the GFP(618)DR3His construct (Fig. 7c).

## Discussion

### Site-Specific Labeling System

The Cy3NTA-based labeling method detailed in this study takes advantage of the well-characterized interaction between Ni^2+^ atoms and imidazole side chains of histidine residues that is used for purification of His-tagged proteins [11]. In this study, Cy3 was coupled to two NTA residues to create Cy3NTA. This compound had a higher apparent binding affinity for binding His tags (0.1–0.5 µM in this study and [12]) compared to mono-NTA fluorophore conjugates which have apparent Kd values between 1–4 µM [13], [18]. In addition, lengthening the His tag from 6 to 10 residues further enhanced binding affinity, most likely by increasing the probability of a productive collision between the NTA/Ni^2+^ group and the His tag.

An advantage of this labeling system is site-specificity. Cy3NTA can be targeted to virtually any desired region of a protein that contains a surface exposed His tag. For RyR1, this method is well suited for site-specific labeling experiments for the following reasons. 1) Nonspecific binding of Cy3NTA to endogenous histidines in RyR1 should be minimal since this protein contains only one segment with two consecutive histidines and no segment longer than 2 [8]. 2) Neither wildtype RyR1 nor GFP-RyR1 lacking a His tag bound to the NTA-agarose column, thus indicating that these proteins lack Ni/NTA binding sites and thus should not be able to bind Cy3NTA. 3) All His-tagged GFP-RyR1 fusion proteins bound to the NTA-agarose column thus indicating that the introduced His_10_ tags are available to bind Ni/NTA and, by extension, Cy3NTA. 4) When His_10_ tags were placed in RyR1 positions predicted to be in close molecular proximity to the N-terminal GFP, a high degree of FRET was observed (Fig. 5–6). This result indicates that Cy3NTA bound to these inserted His_10_ tags in RyR1.

An additional advantage of this labeling system is reversibility. After making a FRET measurement, the bound NTA-fluorophore can be removed with either EDTA or imidazole and a new NTA-fluorophore can potentially be applied. This approach has been used with NTA conjugated to the fluorescent dye, Atto-647 to examine diffusion of His_10_-tagged serotonin receptors in the plasma membrane of HEK-293 cells [18]. In the FRET-based assay system developed in the present study, a second fluorescence acceptor with a different R_0_ value could be added to calibrate distance measurements with Cy3NTA.

Finally, this labeling system provides a high degree of versatility due to the relative ease in construction of new NTA-based probes. Numerous such probes have already been created including NTA coupled with either quantum dots, conformationally sensitive fluorescent probes, biotin or numerous fluorophores with varying spectral properties [14]–[16], [19], [20]. Thus, His-tagged RyRs can be potentially probed with novel NTA-based fluorophores for numerous downstream applications.

### FRET System

In this study, GFP was chosen as a donor fluorophore in the FRET measurements due to its high quantum yield and lack of susceptibility to quenching via either collisional or static mechanisms [21]. The N-terminal domain of RyR1 was selected as a site to attach the GFP donor because previous studies have indicated that GFP fusion at this site does not affect RyR1 activity [3], [22], [23]. In addition, the N-terminal domain is located in the “clamp” domain in the corners of the RyR1 homotetramer approximately 19.7 nm from each other [22], a distance too far for cross-FRET between RyR subunits to occur using the GFP-Cy3 FRET pair. GFP was fused to RyR1 using flexible glycine-rich linkers to permit free rotation of the GFP chromophore, which is an important consideration in FRET measurements that require randomization of donor and acceptor fluorescence dipoles.

There is little question that GFP fused to RyR1 is quenched by Cy3NTA via FRET due to the fact that: 1) The GFP chromophore is shielded within a “beta-can” structure [21] that should prevent quenching of GFP fluorescence by Cy3 via non-FRET mechanisms. 2) While binding of Ni^2+^ to the His_10_ tags could potentially quench GFP fluorescence via FRET [24], [25], this is unlikely because neither Ni^2+^ alone nor Ni^2+^ complexed with NTA quenched GFPHis_6_ fluorescence (Fig. 2b). 3) In the cell-based FRET measurements, GFP-RyR1 (N-term His) fluorescence recovered after photobleaching Cy3 (Fig. 5). This technique is commonly used to quantify FRET in systems employing donor and acceptor fluorescent proteins [26]. The recovery of donor fluorescence after photobleaching of the acceptor is strong evidence that Cy3NTA-dependent decreases in GFP fluorescence occurs via FRET.

### FRET Measurements in Intact RyRs

Equal levels of FRET (∼75%) were observed between the donor GFP fused to the N-terminus of RyR1 and the Cy3NTA acceptor bound to His tags introduced either at the N-terminus of GFP or between GFP and RyR1 (Fig. 6b). This finding is consistent with the fact that the N- and C-termini of GFP (where the His_10_ tags were introduced) are located on the same face of the GFP beta can [21] and thus are approximately the same distance from the GFP donor chromophore. This result provides strong evidence that the level of FRET is dependent on the proximity between the donor and acceptor chromophores introduced into RyR1.

FRET was also observed between GFP at position 618 and Cy3NTA bound to a His_10_ tag adjacent to divergent region 3 at amino acid position 1861. This level of FRET (53%) was higher than the level of FRET observed (20%) between GFP at position 1 and Cy3NTA bound to the His_10_ tag at DR3. These findings are consistent with predicted levels of FRET between these points estimated from cryo EM maps localizing the N-terminus, amino acid 423 and amino acid 1903 [3], [22], [27], positions roughly analogous to the locations of GFP (amino acid position 1, 618) and the His_10_ tag (position 1861) in the present study. The estimated distance between residue 423 and 1903 of 6.3 nm is ∼2 nm closer than the estimated distance between the N-terminus and amino acid residue 1903. The FRET measurements in this study also follow this predicted trend in that a higher level of FRET is observed between donor and acceptor molecules placed at amino acid residues 618 to 1861 compared to these molecules placed at residues 1 and 1861. Conversion of these FRET levels to distances would probably be inaccurate due to the physical dimensions of the donor (GFP: 3×4.5 nm) and acceptor (Cy3NTA: 3×1.8 nm). However, the fact that the level of FRET increases as predicted when GFP is placed closer to the DR3 His_10_ tag coupled with the observation that FRET is being measured across a distance of over 1000 amino acid residues in the RyR1 primary sequence demonstrates the utility of this technique in structural mapping of the RyR.
